# Supplementary material for: Tuning ultrasmall theranostic nanoparticles for MRI contrast and radiation dose amplification
Source: Theranostics. 2023 Aug 21;13(14):4711–29. doi: 10.7150/thno.85663 (PMC10526655; doi:10.7150/thno.85663)
Supplement: Supplementary file 1 — Supplementary figures. [file thnov13p4711s1.pdf]

Supplementary Material

Tuning ultrasmall theranostic nanoparticles for MRI contrast and radiation dose amplification

Needa Brown[#], Paul Rocchi[#], Léna Carmès, Romy Guthier, Meghna Iyer, Léa Seban, Toby Morris, Stephanie Bennett, Michael Lavelle, Johany Penailillo, Ruben Carrasco, Chris Williams, Elizabeth Huynh, Zhaohui Han, Evangelia Kaza, Tristan Doussineau, Sneh M. Toprani, Xingping Qin, Zachary D. Nagel, Kristopher A. Sarosiek, Agnès Hagège, Sandrine Dufort, Guillaume Bort, François Lux, Olivier Tillement, and Ross Berbeco[*]*

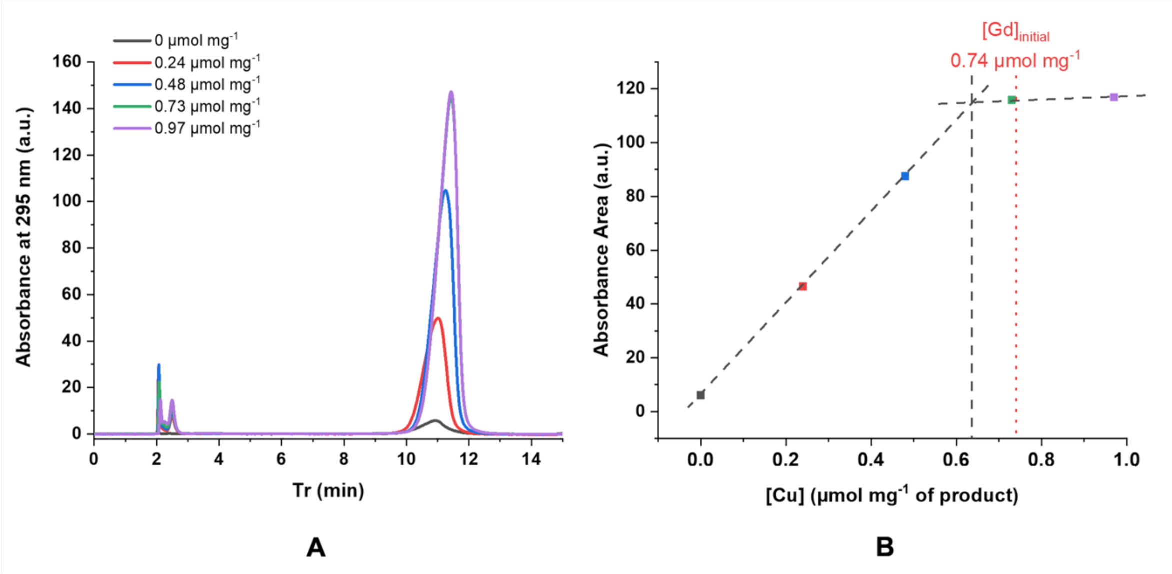

Figure S1: Measurement of free DOTAGA at the beginning of the bismuth chelation. **A)** Chromatograms of samples with increasing amount of Cu^{2+}. The increase in absorbance at 295 nm is due to the formation of DOTAGA@(Cu^{2+}). **B)** Measured area depends on the amount of Cu^{2+}. The slope break shows the amount of free DOTAGA in the product.

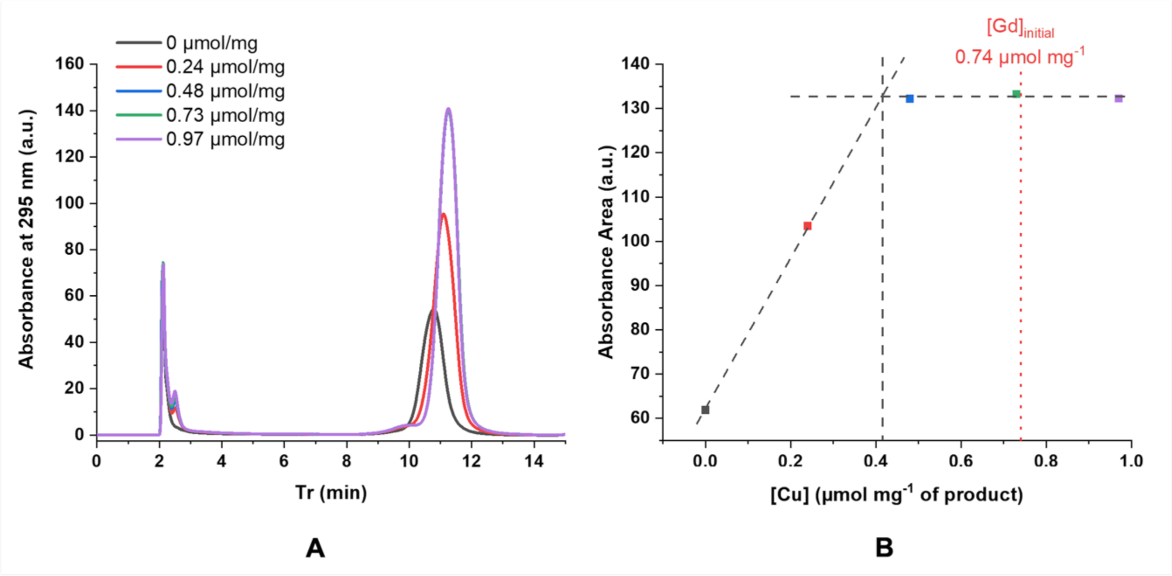

Figure S2: Measurement of free DOTAGA after the first chelation bismuth chelation of 30%. **A)** Chromatograms of samples with increasing amount of Cu^{2+}. The increase in absorbance at 295 nm is due to the formation of DOTAGA@(Cu^{2+}). **B)** Measured area depends on the amount of Cu^{2+}. The slope break shows the amount of free DOTAGA in the product.

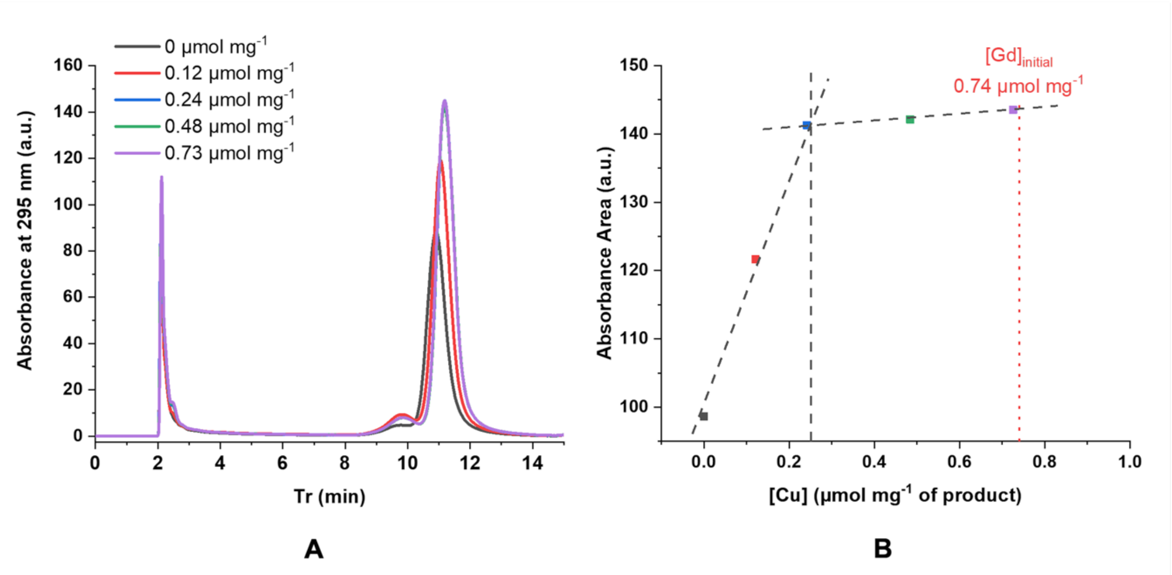

Figure S3: Measurement of free DOTAGA after the second chelation bismuth step of 20%. **A)** Chromatograms of samples with increasing amount of Cu^{2+}. The increase in absorbance at 295 nm is due to the formation of DOTAGA@(Cu^{2+}). **B)** Measured area depends on the amount of Cu^{2+}. The slope break shows the amount of free DOTAGA in the product.

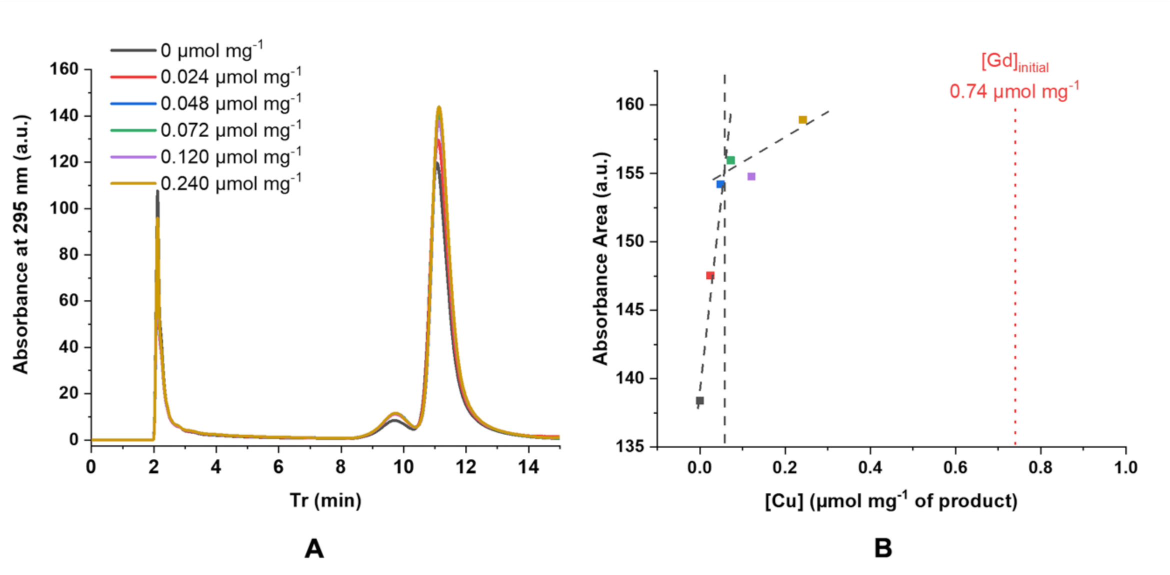

Figure S4: Measurement of free DOTAGA after the third chelation bismuth step of 20%. **A)** Chromatograms of samples with increasing amount of Cu^{2+}. The increase in absorbance at 295 nm is due to the formation of DOTAGA@(Cu^{2+}). **B)** Measured area depends on the amount of Cu^{2+}. The slope break shows the amount of free DOTAGA in the product.

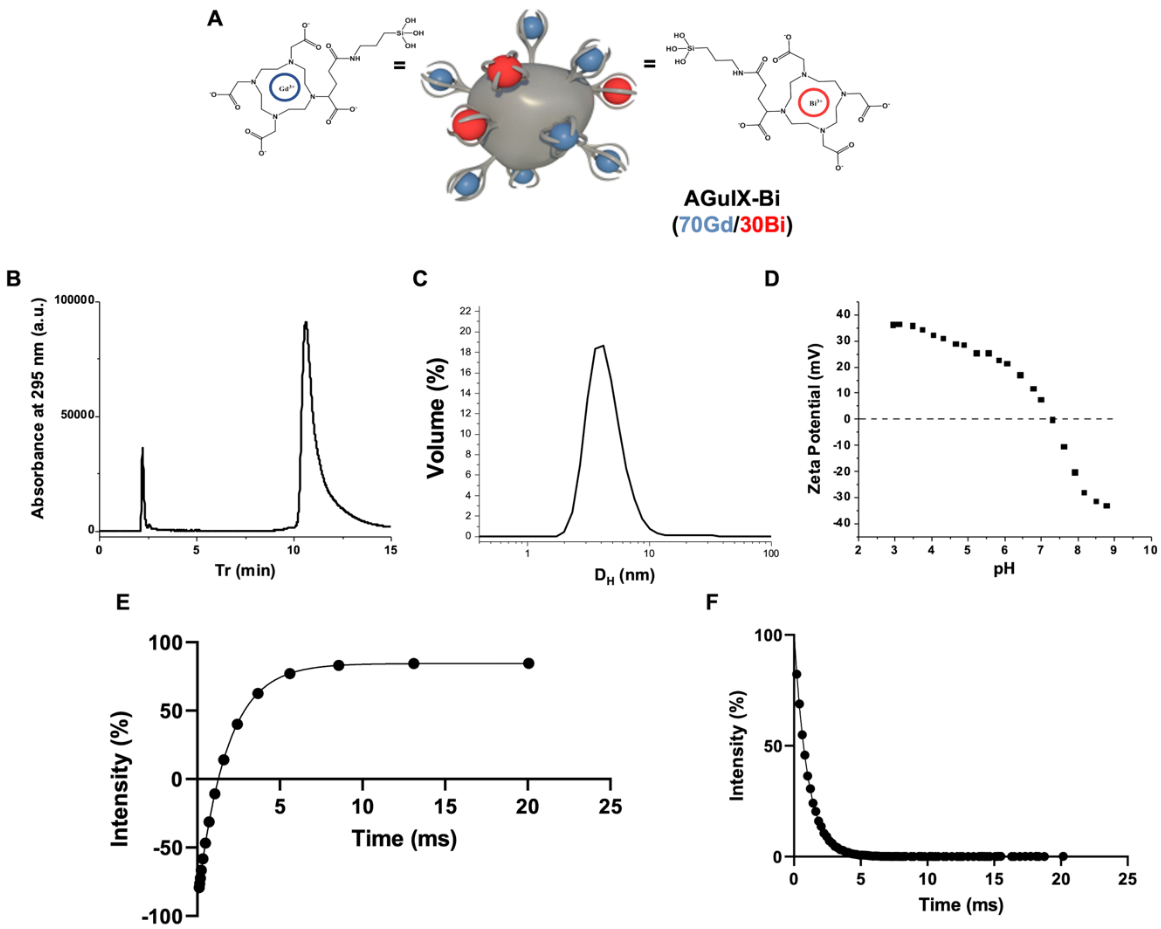

Figure S5: A) Schematic representation of AGuIX-Bi (70Gd/30Bi) NP with detailed structure of chelated DOTAGA groups. **B)** HPLC-UV chromatograms of AGuIX-Bi (70Gd/30Bi) NP (10 µL, 10 g L^{-1}) recorded at 295 nm. **C)** Hydrodynamic diameter distribution in volume obtained by dynamic light scattering. **D)** Zeta potential vs pH for AGuIX-Bi (70Gd/30Bi) NP. Relaxivity measurements for **E)** r_1 and **F)** r_2 at 1.5 T of AGuIX-Bi (70Gd/30Bi) NP.

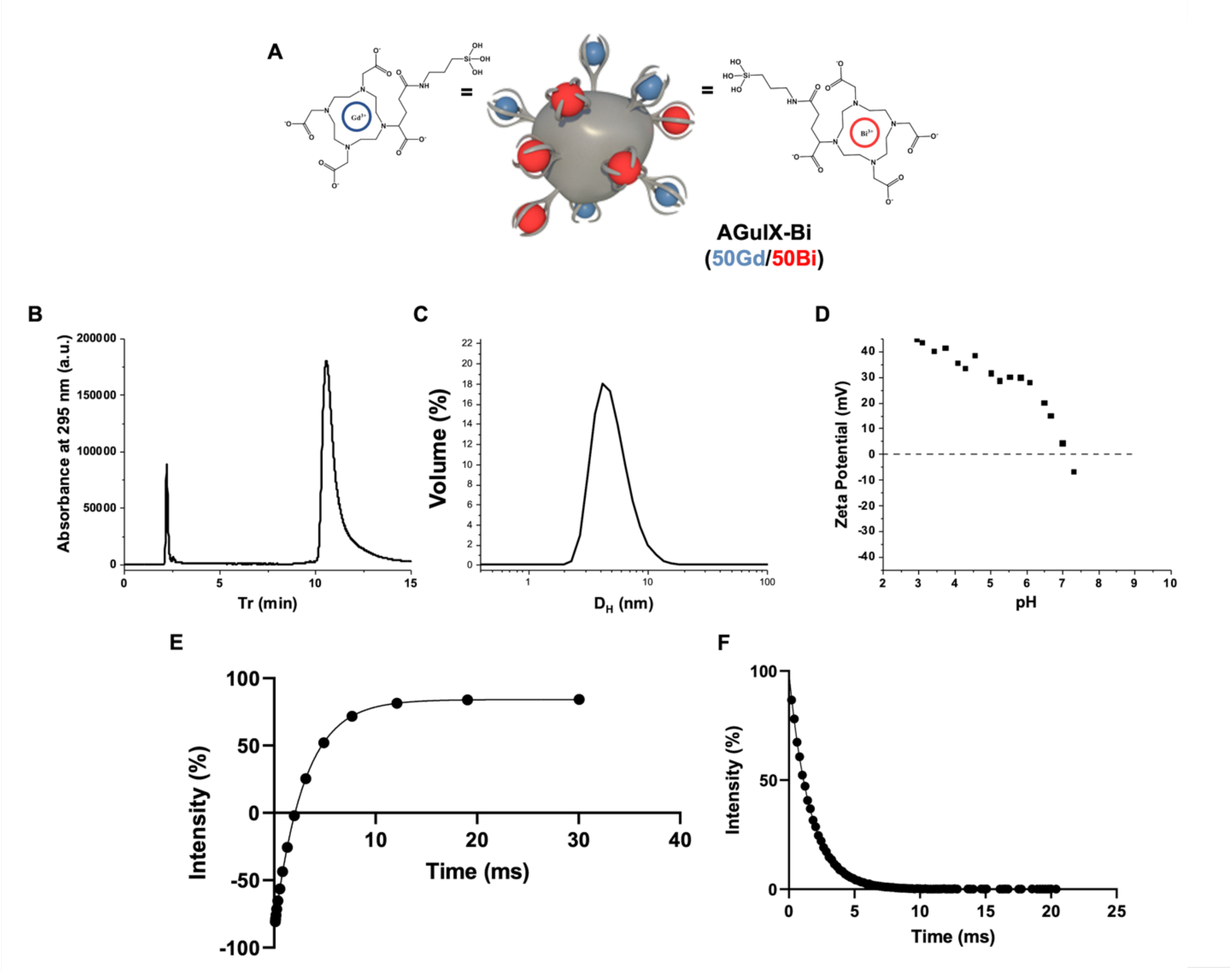

Figure S6: A) Schematic representation of AGuIX-Bi (50Gd/50Bi) NP with detailed structure of chelated DOTAGA groups. **B)** HPLC-UV chromatograms of AGuIX-Bi (50Gd/50Bi) NP (10 µL, 10 g L^{-1}) recorded at 295 nm. **C)** Hydrodynamic diameter distribution in volume obtained by dynamic light scattering. **D)** Zeta potential vs pH for AGuIX-Bi (50Gd/50Bi) NP. Relaxivity measurements for **E)** r_1 and **F)** r_2 at 1.5 T of AGuIX-Bi (50Gd/50Bi) NP.

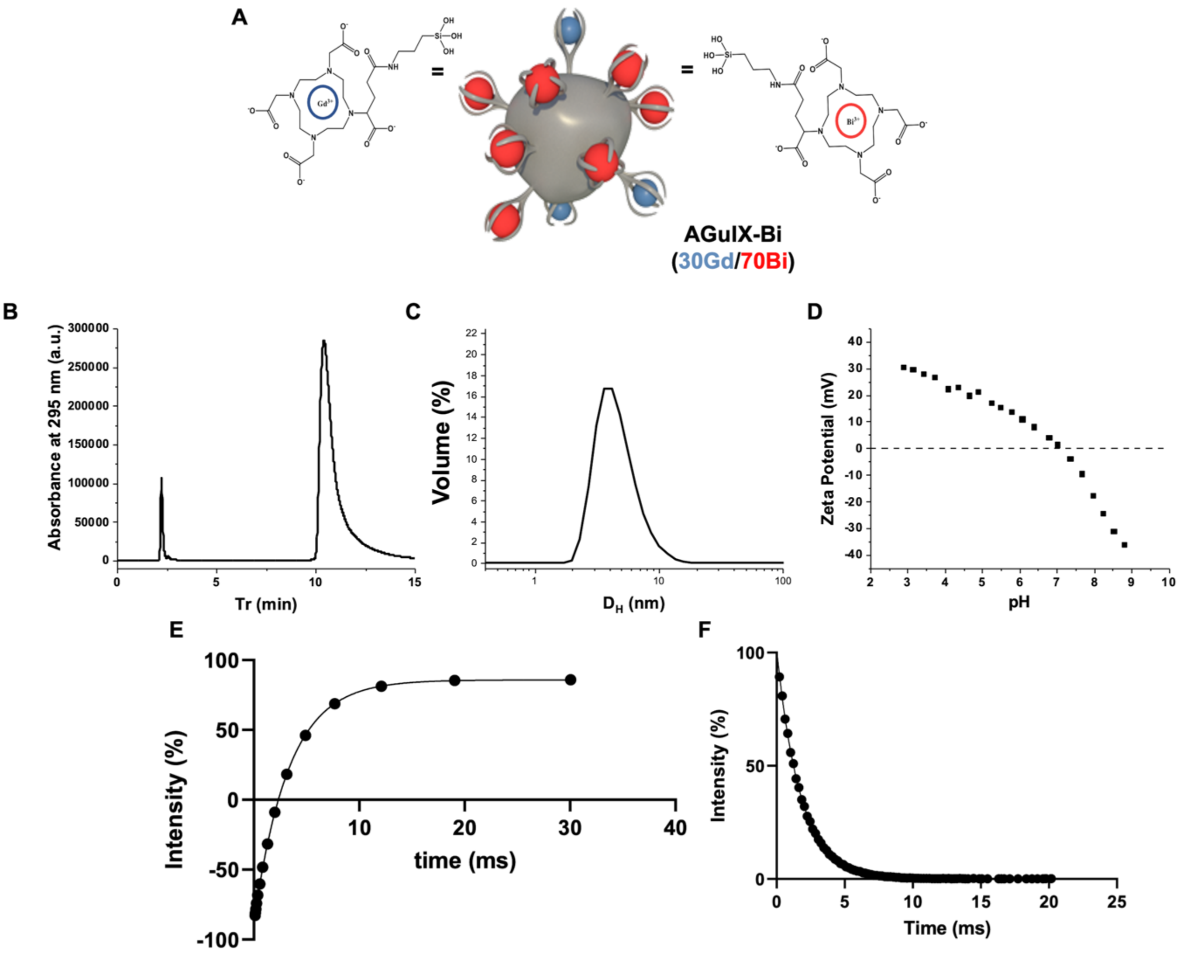

Figure S7: A) Schematic representation of AGuIX-Bi (30Gd/70Bi) NP with detailed structure of chelated DOTAGA groups. **B)** HPLC-UV chromatograms of AGuIX-Bi (30Gd/70Bi) NP (10 µL, 10 g L^{-1}) recorded at 295 nm. **C)** Hydrodynamic diameter distribution in volume obtained by dynamic light scattering. **D)** Zeta potential vs pH for AGuIX-Bi (30Gd/70Bi) NP. Relaxivity measurements for **E)** r_1 and **F)** r_2 at 1.5 T of AGuIX-Bi (30Gd/70Bi) NP.

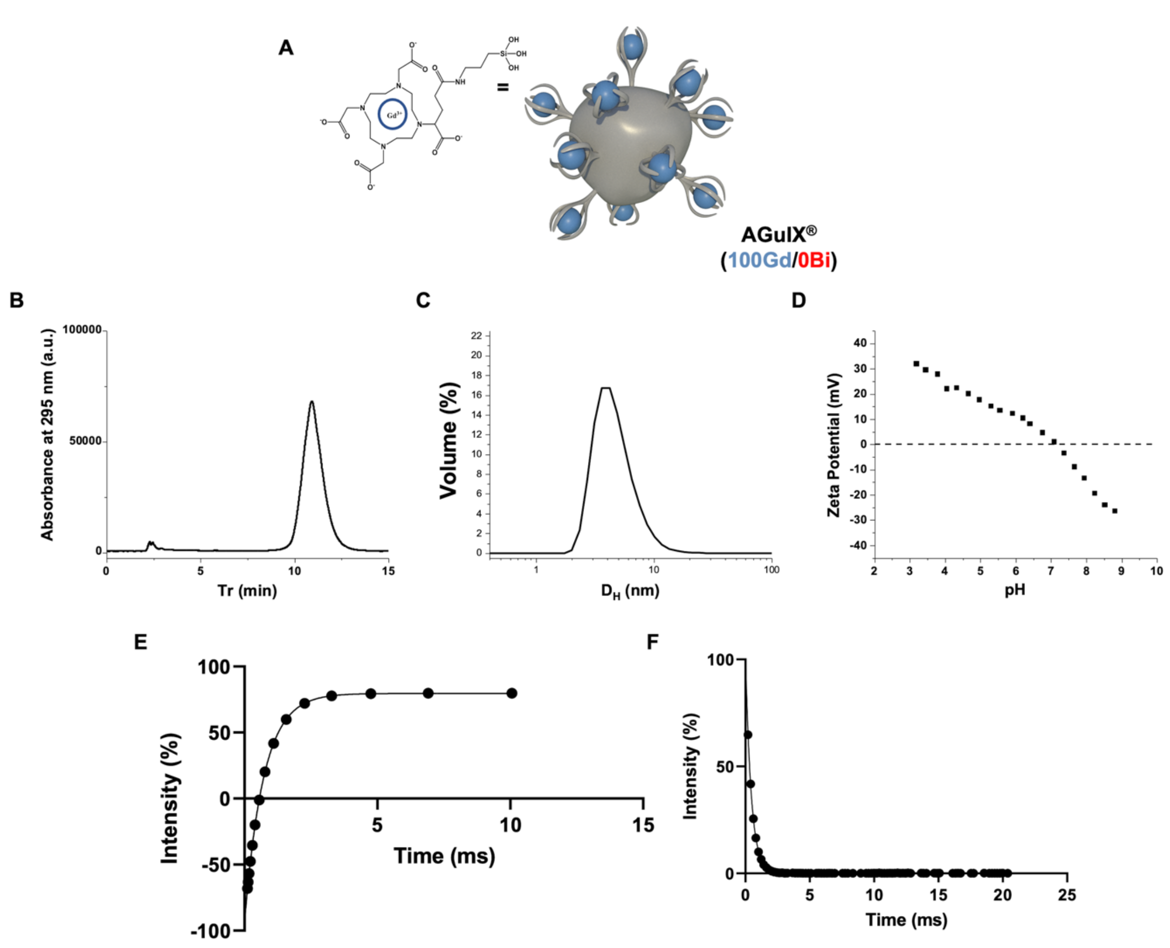

Figure S8: A) Schematic representation of AGuIX® NP with detailed structure of chelated DOTAGA groups. **B)** HPLC-UV chromatograms AGuIX® NP (10 µL, 100 g L^{-1}) recorded at 295 nm. **C)** Hydrodynamic diameter distribution in volume obtained by dynamic light scattering. **D)** Zeta potential vs pH for AGuIX® NP. Relaxivity measurements for **E)** r_1 and **F)** r_2 at 1.5 T of AGuIX® NP.

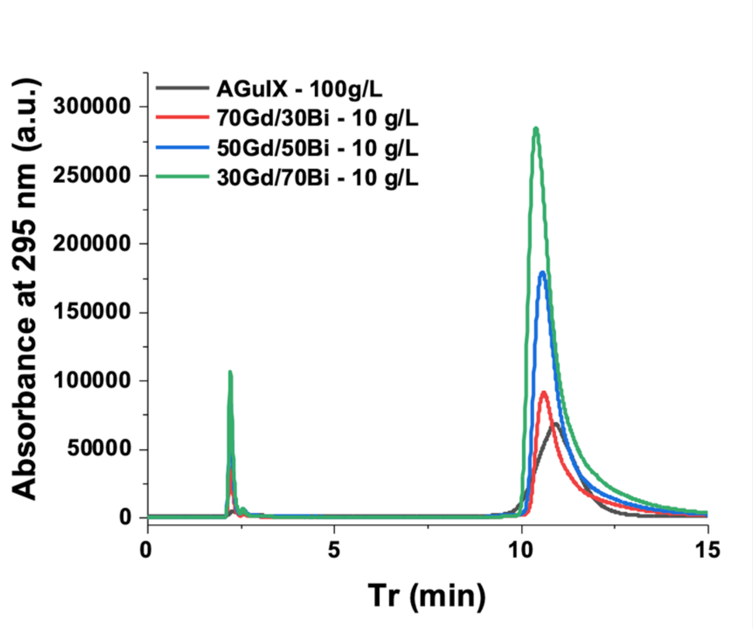

Figure S9: HPLC-UV chromatograms of AGuIX® particle (10 µL, 100 g L^{-1}) and the three final Gd/Bi (10 µL, 10 g L^{-1}) recorded at 295 nm. The increase of the absorbance at the level Tr is due to the creation of DOTAGA(Bi).

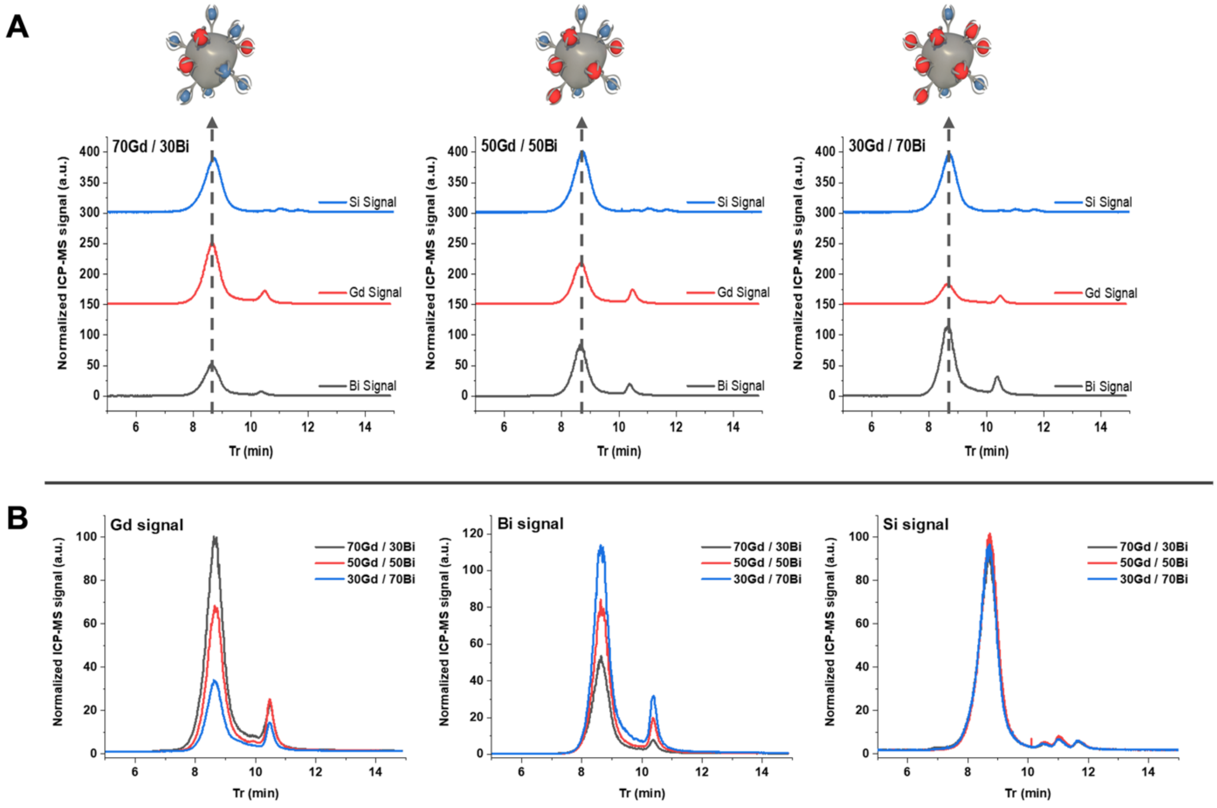

C

Product	Area Gd	Area Bi	Area Si
AGuIX-Bi (70Gd/30Bi)	100	46.5	95.4
AGuIX-Bi (50Gd/50Bi)	71.6	69.9	100
AGuIX-Bi (30Gd/70Bi)	42	100	96.4

Figure S10: A) HPLC-SEC-ICP-MS chromatograms of the three final Gd/Bi following Gd, Bi and Si signals. We can observe the elemental ratio evolution between each product. **B)** HPLC-SEC-ICP-MS chromatograms of the three final Gd/Bi regrouped by specific element **C)** showing the ratio evolution of each measured element.

	AGuIX®	20% Gd 0% Bi	20% Gd 30% Bi	20% Gd 70% Bi
**Isoelectric point**	**7.15**	**5.58**	**6.63**	**6.95**

Figure S11: IEP evolution all along the process. The decrease from one step to another is related to the generation of DOTAGA groups at the surface. In the contrary, the increase of the IEP is related to the chelation of a free DOTAGA by Gd^{3+} or Bi^{3+}.

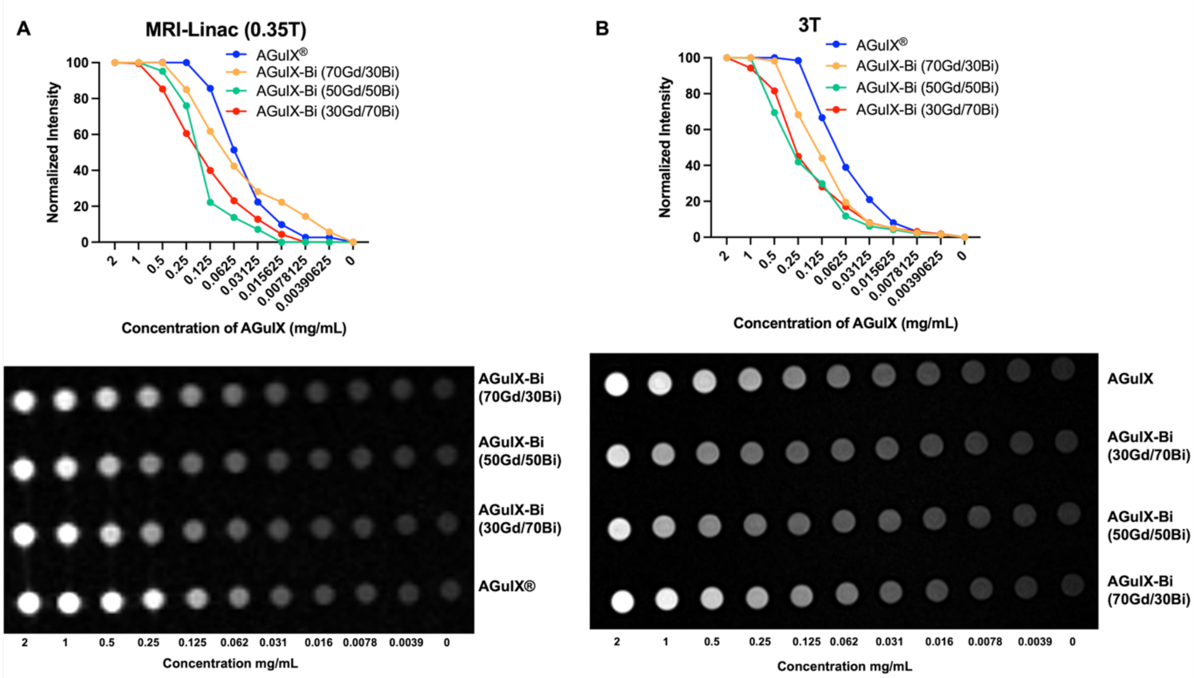

Figure S12: Agar phantom MRI images and signal intensity quantification of AGuIX® and various ratios of AGuIX-Bi nanoparticles in a **A)** 0.35 T MRIdian MRI-Linac and **B)** 3 T Siemens MR scanner. Signal intensity was normalized to highest concertation of nanoparticle.

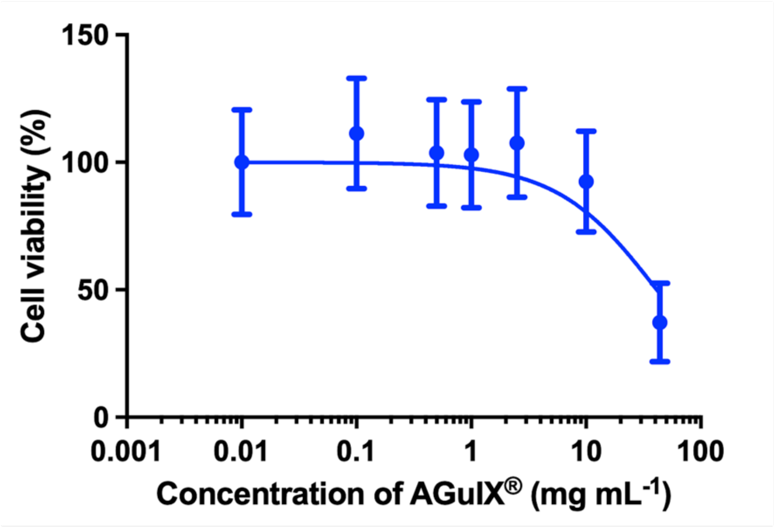

Figure S13: *In vitro* cytotoxicity of AGuIX® as a function of concentration 24 h post-incubation in a NSCLC A549 murine model (n = 3).

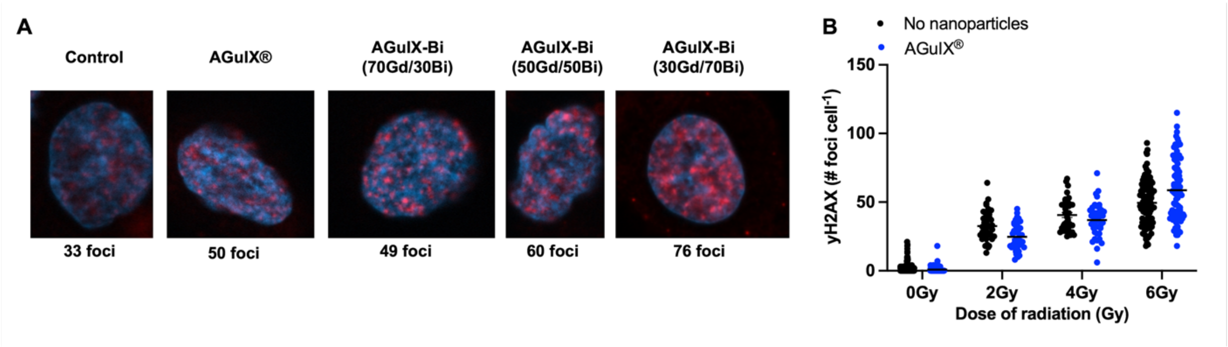

Figure S14: A) Representative single cell nuclei (blue) showing increasing γH2AX foci (red dots) with increasing ratio of Bi. **B)** Breaks in dsDNA following radiation was assayed using γH2AX for AGuIX® (n = 60 - 80 cells).

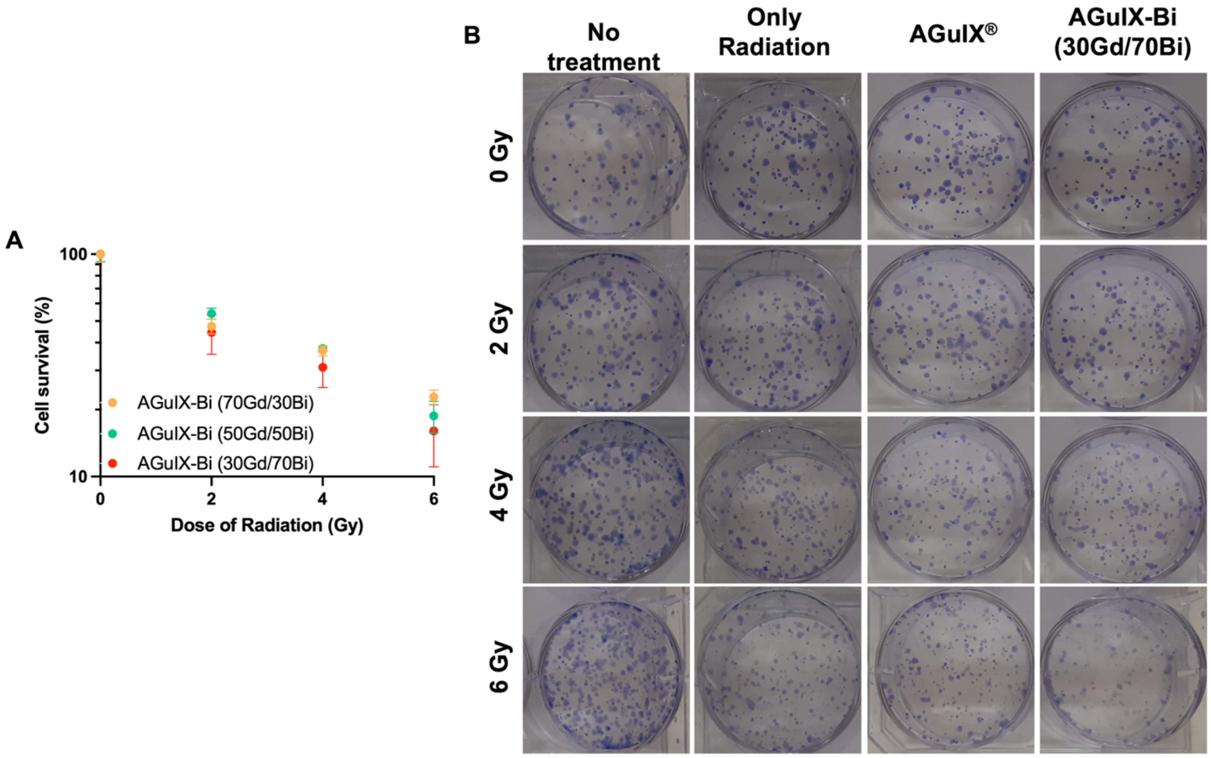

Figure S15: A) Clonogenic assay comparing the impact of increasing Bi ratio on cell survival. **B)** Clonogenic assay representative images.

F

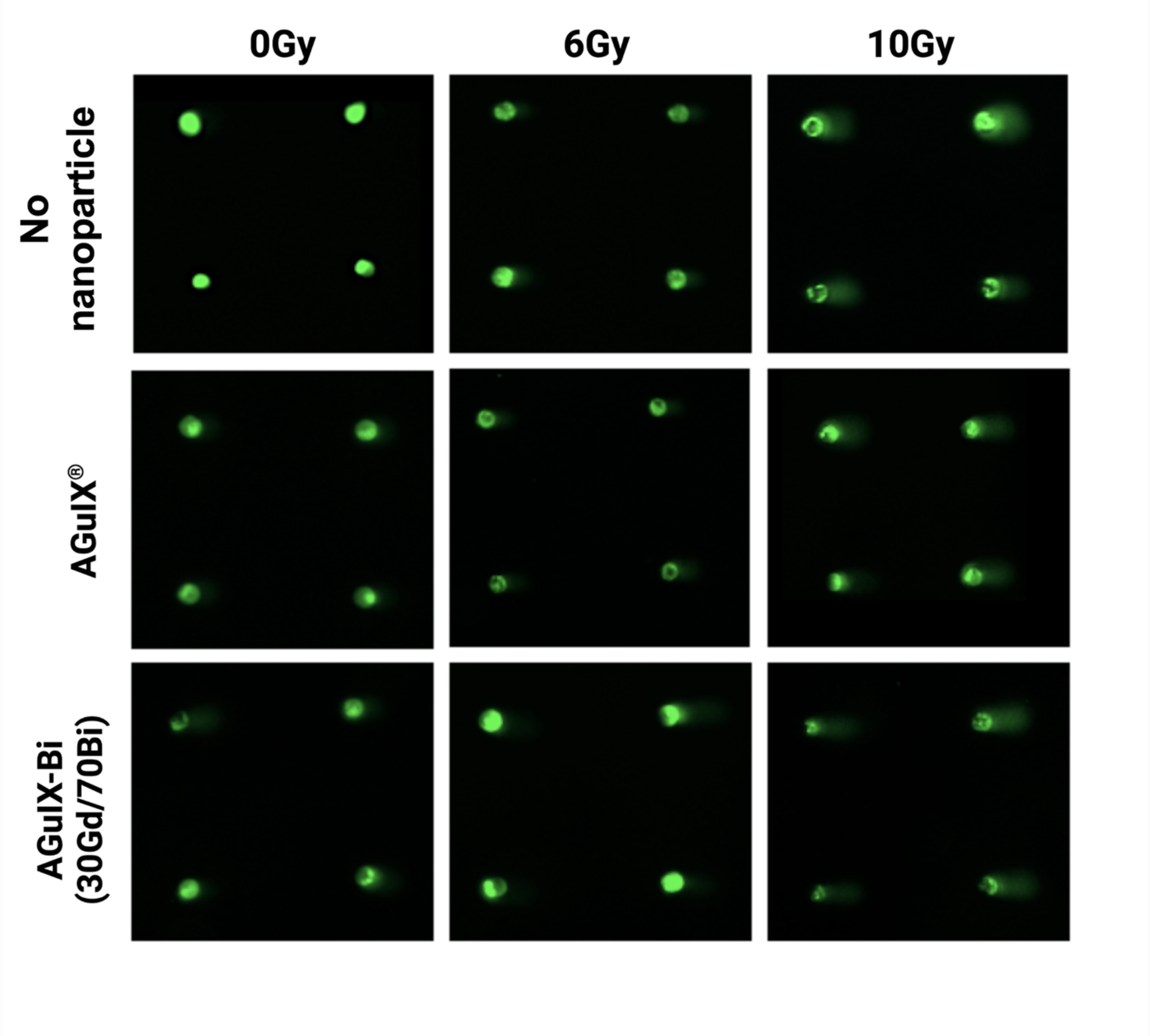

Figure S16: Representative comets from A549 cells treated with AGuIX® or AGuIX-Bi (30Gd/70Bi) with sham controls or irradiated at 6 Gy or 10 Gy.

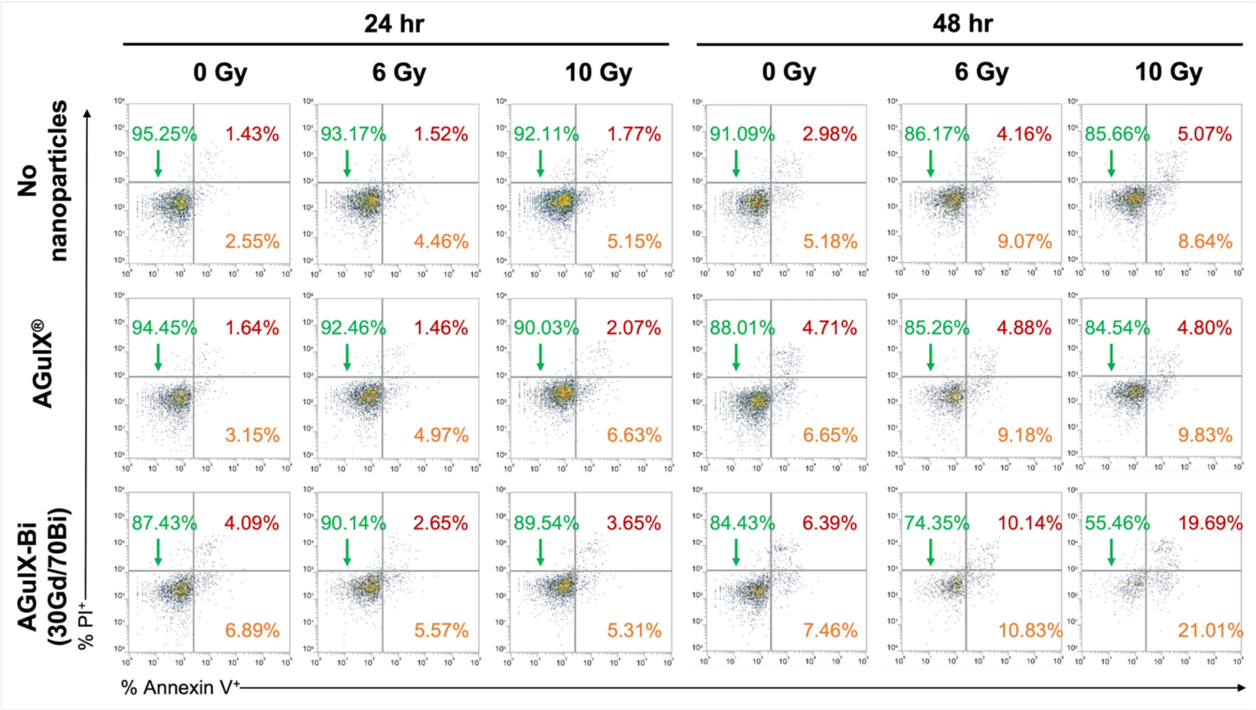

Figure S17: Representative flow cytometry data of cells positive for annexin V (AxV, orange), propidium iodine (PI, green), or AxV+PI+ (red). A549 cells treated with AGuIX® or AGuIX-Bi (30Gd/70Bi) with sham controls or irradiated at 6 Gy or 10 Gy.

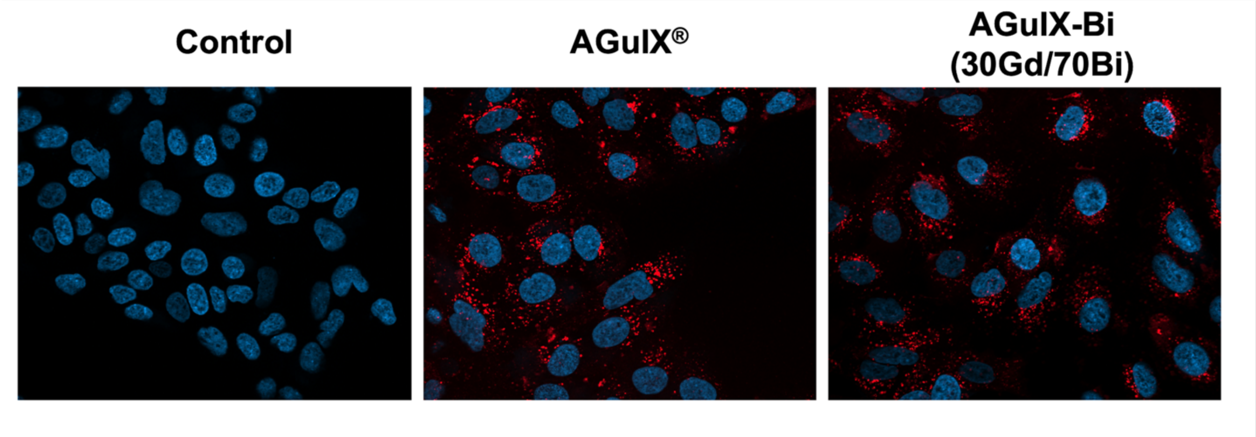

Figure S18: Representative nanoparticle (red) internalization images of A549 cells (blue) treated with either AGuIX® or AGuIX-Bi (30Gd/70Bi) for 24 h.

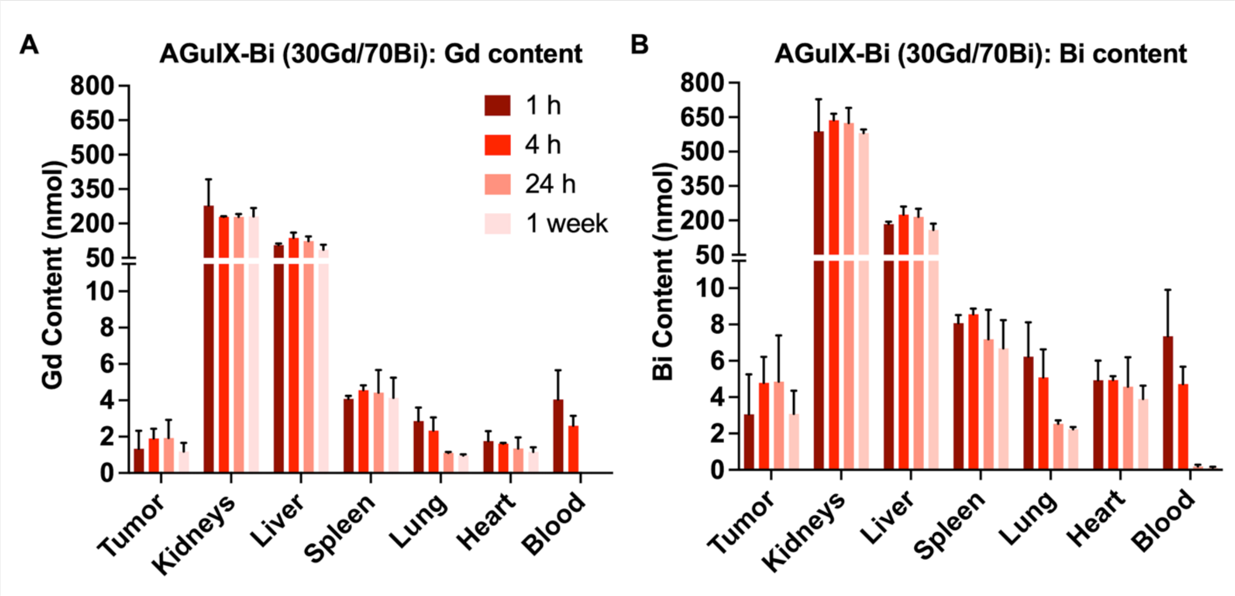

Figure S19: Raw ICP-MS nanomolar amount of **A)** gadolinium and **B)** bismuth in extracted tissue from mice treated with AGuIX-Bi (30Gd/70Bi). An average Bi/Gd molar ratio of 2.3 ± 0.56 was observed across all extracted tissue.

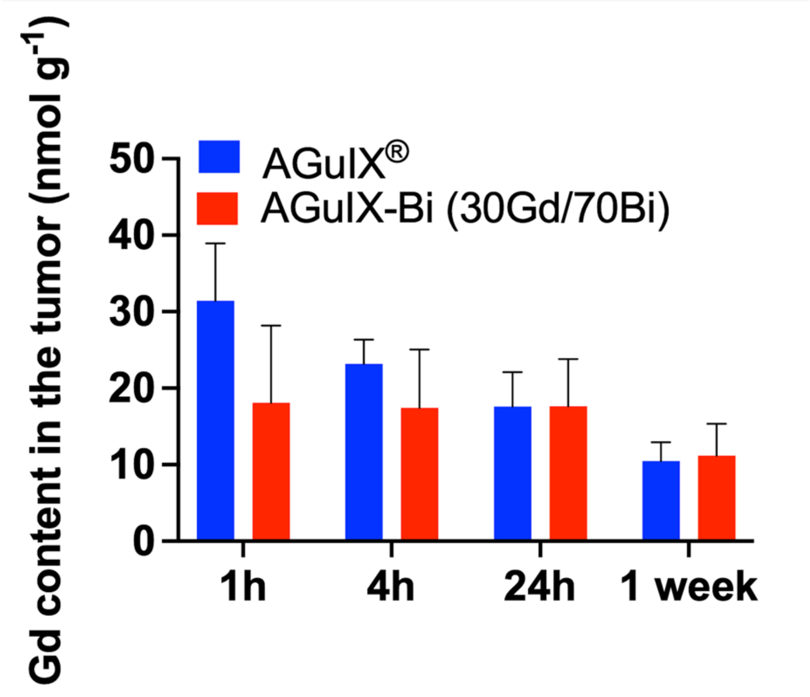

Figure S20: Raw ICP-MS nanomolar amount of gadolinium in extracted tumor tissue.

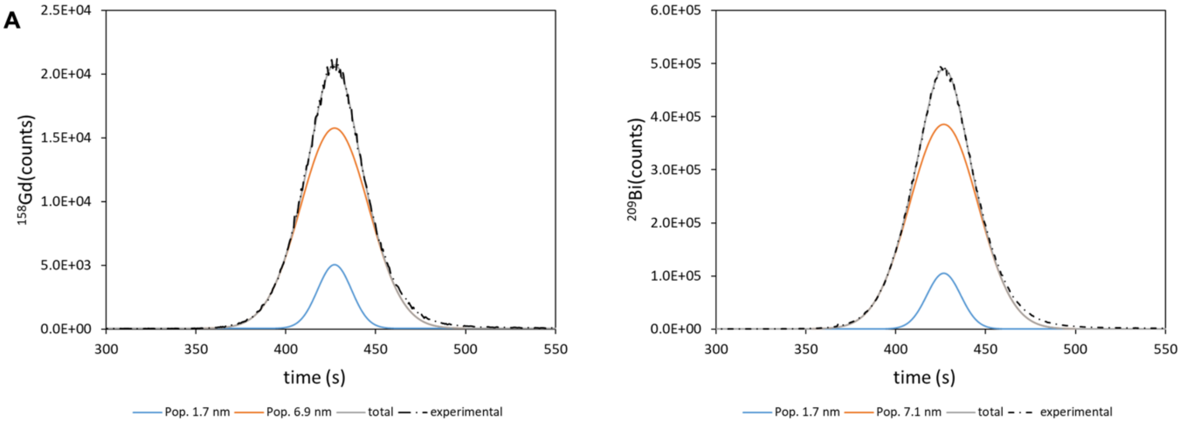

B

**Signal**	**Pop 1** (nm)	**Metal proportion in Pop 1** (%)	**Pop 2** (nm)	**Metal proportion in Pop 2** (%)
^{158}Gd	7.1 ± 0.3	86 ± 1	1.7 ± 0.1	14 ± 1
^{209}Bi	7.1 ± 0.5	87 ± 2	1.8 ± 0.4	13 ± 2

Figure S21. A) Representative taylorgram and corresponding bimodal fit for gadolinium (left) and bismuth (right) signal for AGuIX-Bi (30Gd/70Bi) nanoparticles. The signal (dashed black line) was fitted with the sum (solid gray line) of two gaussian curves (solid blue and orange lines) of the two populations within the sample. **B)** Average hydrodynamic diameters and Gd and Bi repartition from the TDA experiments (n = 3).

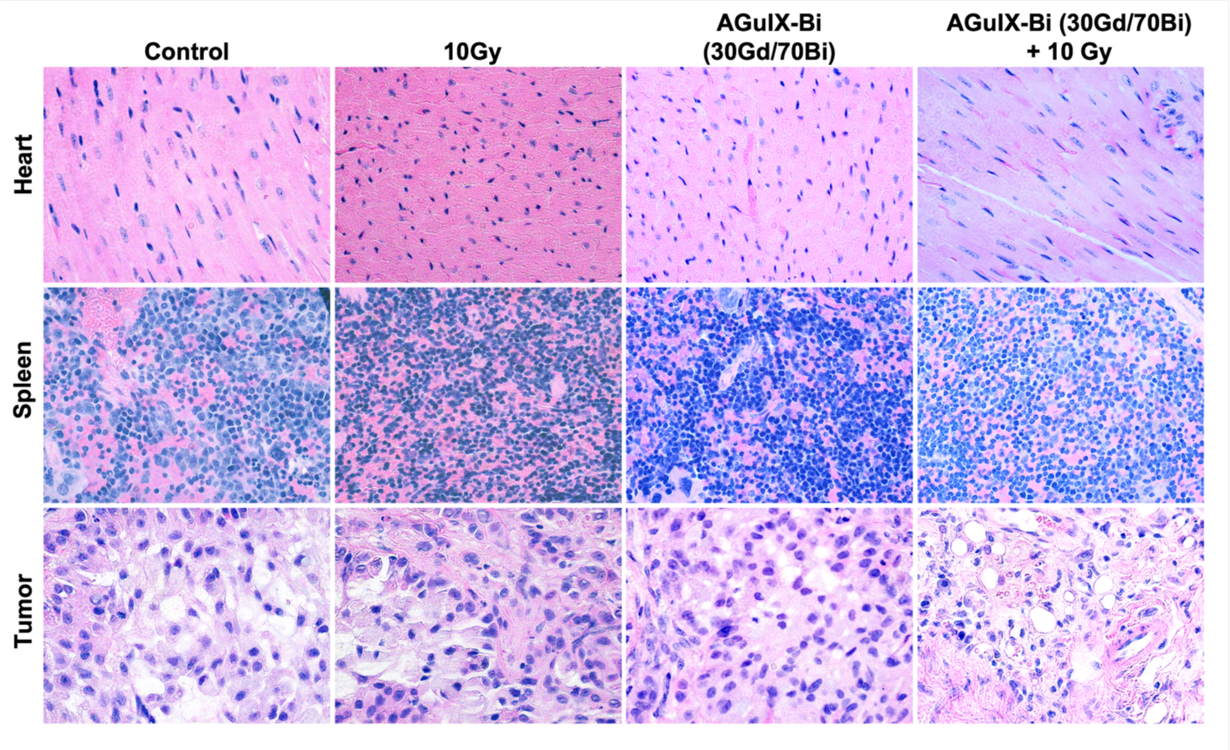

Figure S22: Histopathology of mice treated with either saline or AGuIX-Bi (30Gd/70Bi) and/or 10 Gy radiation 24 h post-treatment. No short-term toxicity was identified in heart, spleen, or tumor.

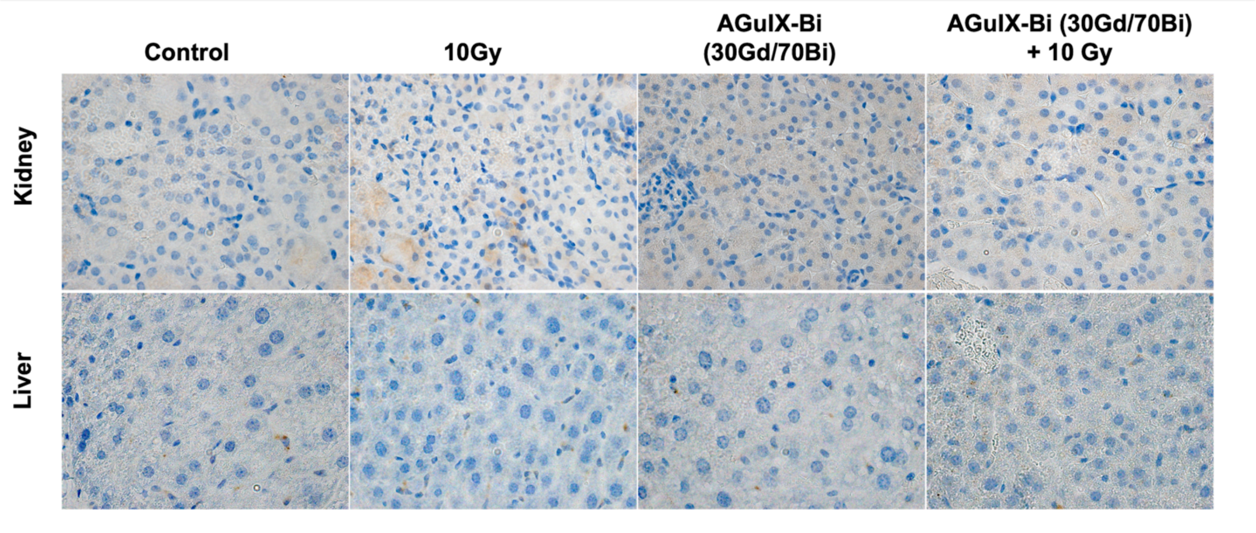

Figure S23: Caspase-3 staining of liver and kidney, which have the highest accumulation of nanoparticles, show no difference in staining.

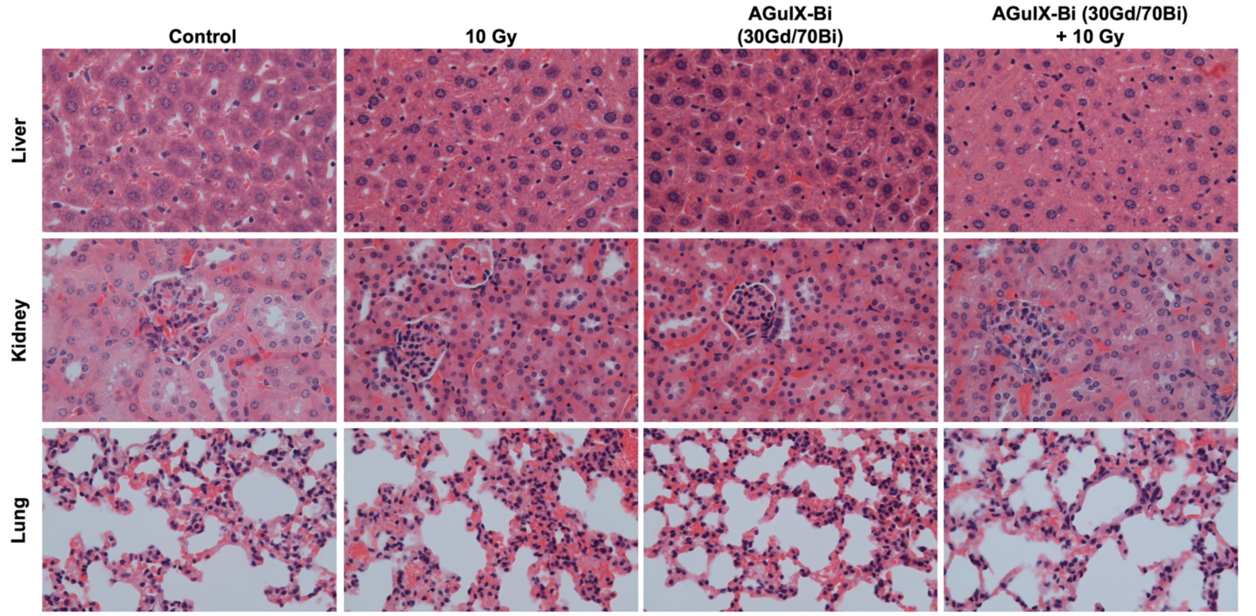

Figure S24: Histopathology of mice treated with either saline or AGuIX-Bi (30Gd/70Bi) and/or 10 Gy radiation 1 mo post-treatment. No long-term toxicity was identified in lungs, kidney, or liver.

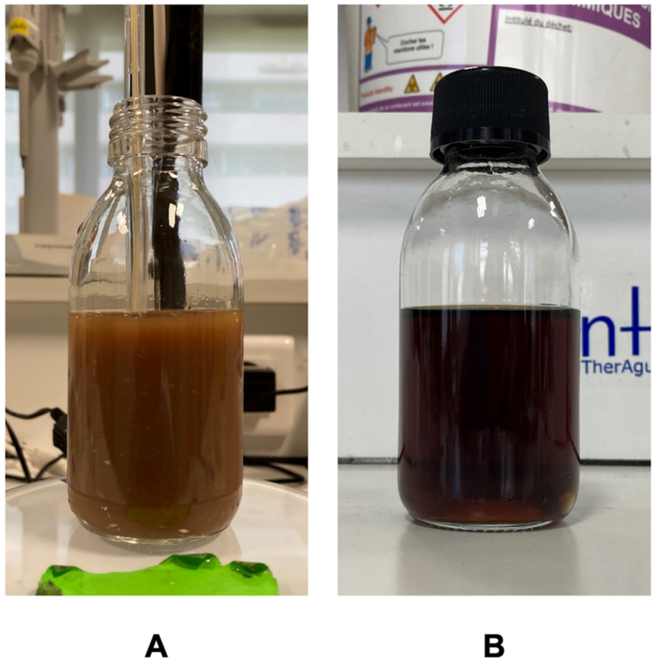

Figure S25: A) Image of the solution after addition of the $BiCl_3$. At high concentrations of $BiCl_3$, the solution precipitates. **B)** Clear solution after pH adjustment and stirring for 48 h at 80 °C.

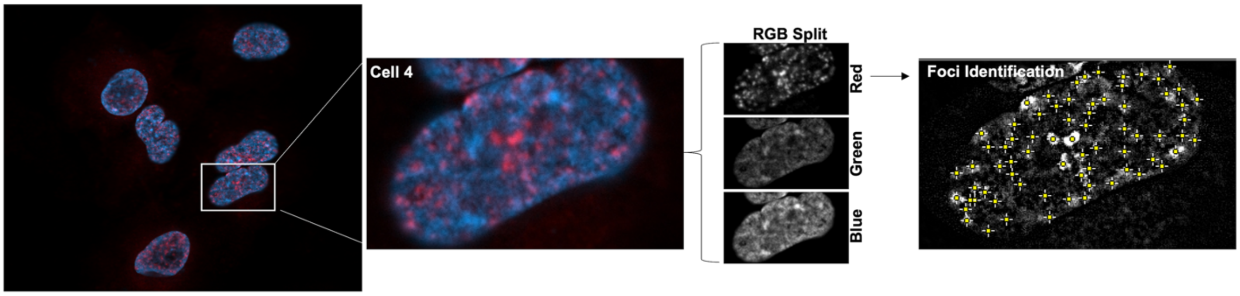

Figure S26: Representative example of ImageJ automated macro steps for γH2AX foci quantification.

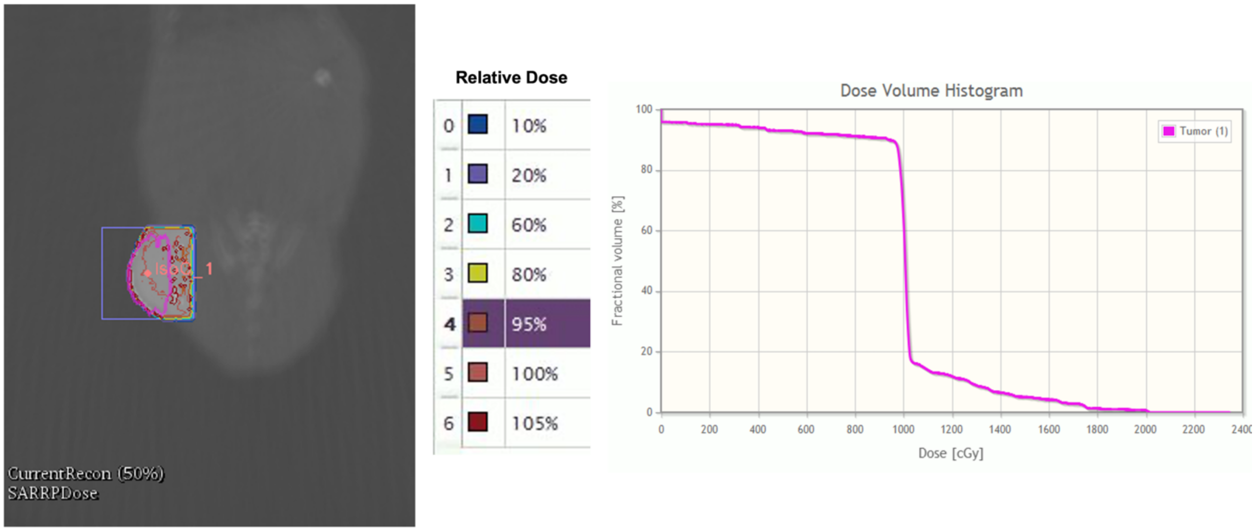

Figure S27: Representative SARRP treatment plan for a mouse given 10 Gy radiation. A cone-beam computed tomography image was acquired at 60 kVp (0.8 mA current with a 1 mm Al filter) to identify tumor isocenter in MuriSlice followed by a single posterior-anterior 220 kVp (13 mA current with a 0.15 mm Cu filter) beam delivering a dose of 10 Gy to a 1 x 1 cm^2 collimated field.

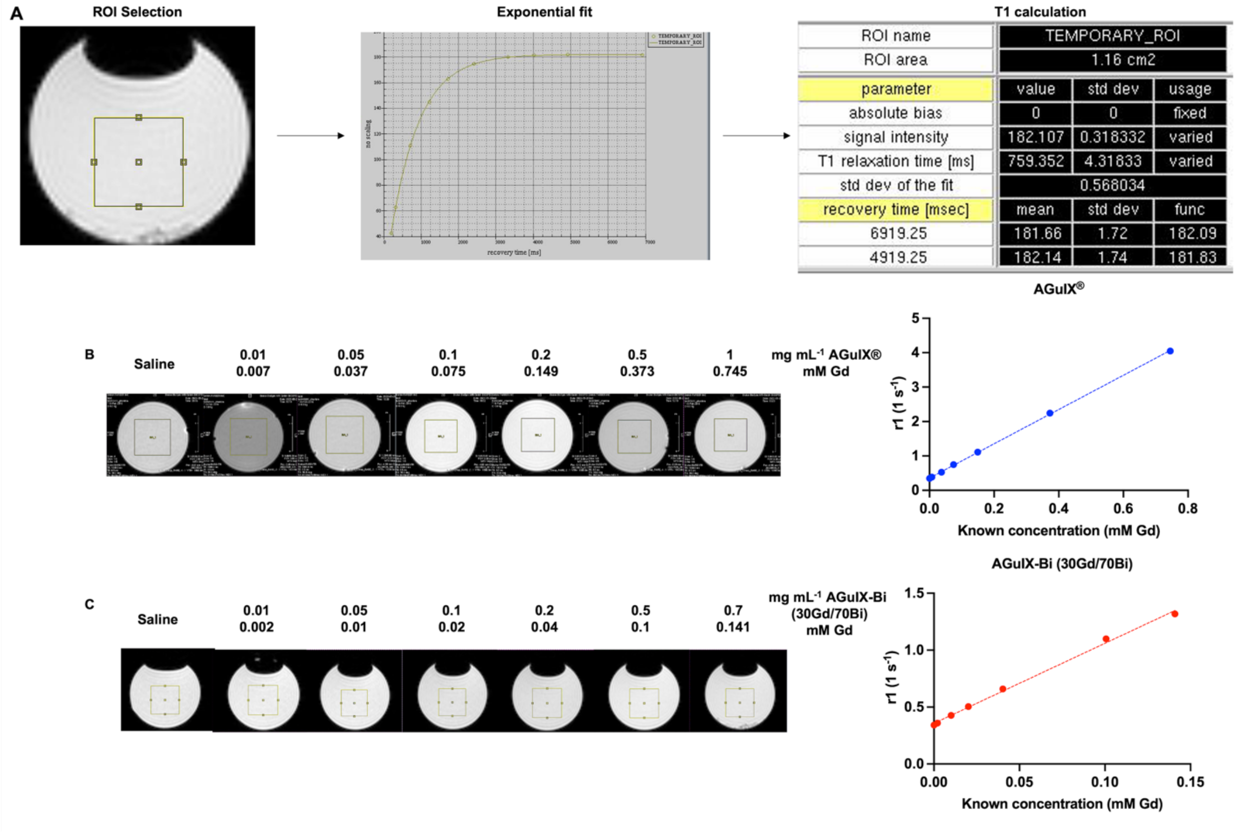

Figure S28: A) Representative example of a phantom of AGuIX-Bi (30Gd/70Bi) used to determine nanoparticle relaxivity at 7 T. Bruker Paravision 6.0.1 was used for MRI data acquisition and T1 map data analysis to determine r_1 for **B)** AGuIX® and **C)** AGuIX-Bi (30Gd/70Bi).
